# Association of MMP9-1562C/T and MMP13-77A/G Polymorphisms with Non-Small Cell Lung Cancer in Southern Chinese Population

**DOI:** 10.3390/biom9030107

**Published:** 2019-03-18

**Authors:** Wen Li, Ming Xi Jia, Jian Hui Wang, Jie Li Lu, Jing Deng, Jian Xin Tang, Cun Liu

**Affiliations:** 1 Key Laboratory of Biological Nanomaterials and Devices, College of Life Sciences and Chemistry, Hunan University of Technology, Zhuzhou 412007, China; liwendream@163.com (W.L.); Mingxijia123@163.com (M.X.J.); lijielu2010@163.com (J.L.L.); Cunliu@aliyun.com (C.L.); 2 National Engineering Laboratory for Rice and Byproducts Deep Processing, College of Food Science and Engineering, Central South University of Forestry and Technology, Changsha 410004, China; 3 School of Chemistry and Bioengineering, Changsha University of Science and Technology, Changsha 410114, China; wangjh0909@csust.edu.cn; 4 Key Laboratory of Advanced Packaging Materials and Technology, College of Packaging and Material Engineering, Hunan University of Technology, Zhuzhou 412007, China

**Keywords:** MMP9, MMP13, single-nucleotide polymorphism, non-small cell lung cancer

## Abstract

Background: Matrix metalloproteinases (MMPs) are capable of degrading and modifying most components of the extracellular matrix (ECM) and the basal membrane (BM), and play crucial roles in cancer invasion and metastasis. MMP gene expressions were regulated primarily at the transcriptional level, which was associated with tumor spread and patient prognosis. Polymorphisms in MMPs have been reported to be associated with non-small cell lung cancer (NSCLC). The objective of this study aim to evaluate the serum levels and polymorphisms of MMP-9 and MMP-13 in non-small cell lung cancer patients compared to normal subjects and their correlation to non-small cell lung cancer histopathology findings in Southern Chinese people. Methods: This case–control study included 245 patients with NSCLC and 258 healthy controls. Genomic DNA was extracted by using DNA extraction kit, genotyping was confirmed by polymerase chain reaction-restriction fragment length polymorphism (PCR-RFLP) and direct DNA sequencing, and serum levels of MMP-9 and MMP-13 were measured by using a specific ELISA, Human Matrix Metalloproteinase Enzyme Immunoassay Kits. Statistical analysis was carried out using the SPSS 23.0 software package. Results: The subjects carrying the TT genotype had a decreased risk of lung cancer in MMP9-1562C/T comparing with the CC genotype (*p* = 0.00, OR = 0.45, 95% CI = 0.29–0.68), and the MMP13-77 AA genotype was associated with a decreased risk of NSCLC by comparing with the GG genotype (*p* = 0.03, OR = 0.56, 95% CI = 0.33–0.94). Moreover, the C allele of MMP9-1562C/T could increase serum level of NSCLC in compared with the A allele (OR = 1.19, 95% CI = 0.75–1.89). Similarly, the AA genotype of MMP13 might be a marker of decreased serum level of lung cancer (OR = 0.76, 95% CI = 0.51–1.14). Conclusions: The results of these analyses underline the support of the notion that the CC genotype of MMP9-1562C/T and GG genotypes of MMP13-77G/A were associated with the increased risk NSCLC, and the serum levels of MMP9 and MMP13 were consistent with the results of the SNP analysis. MMP13 and MMP9 might be function as a key oncogene in NSCLC with a Southern Chinese population. Combined detection of SNP and enzyme activity between MMP9 and MMP13 are expected to be a potential diagnostic method of non-small cell lung cancer.

## 1. Introduction

Lung cancer is the leading cause of cancer-related death in many countries, and remains the most common cancer in the world; both in term of new cases and deaths because of the high case fatality [1]. The incidence and mortality patterns of lung cancer closely follow each other at the global level, currently. In the US and the UK, advanced technology and awareness programs have helped decrease the mortality from lung cancer; but this status is not the case in India and Egypt [2]. Epidemiological studies have demonstrated tobacco smoking as well as exposure to environmental tobacco smoke in healthy nontobacco users as the major risk factor for lung cancer [3]. However, some researches demonstrated that lung cancer result from interactions between genetic susceptibility of the individual and risk factors in the environment and a growing number of researches suggest that the incidence of lung cancer is closely related to genetic factors [2,3,4].

As a family of zinc-ion-dependent endopeptidases, more than 26 subtypes of matrix metalloproteinases (MMPs) have been detected in human, currently [5,6]. Matrix metalloproteinases are capable of degrading and modifying most components of the extracellular matrix (ECM) and the basal membrane (BM), and play crucial roles in cancer invasion and metastasis [7,8,9]. Among the MMPs, matrix metalloproteinases-9 (MMP-9), located at human chromosome 20q12-13, is one of the most important enzymes to breakdown extracellular matrix, which plays a crucial role in various types of cancer as a member of MMPs family [9].

Matrix metalloproteinase 13 (MMP13), also named collagenase, is involved in the degradation of collagen fibrillar types I, II, III, and VII and in fast extracellular matrix remodeling [10]. The MMP-13 gene is located in chromosome 11q22 spanning approximately 12.5 kb, and consists of ten exons and nine introns [11]. Among the polymorphisms, single-nucleotide polymorphisms, (SNPs) constituting substitutions of single bases, have been found in the promoter regions of MMP9 and MMP13 genes. One of the most significant of these SNPs is MMP9 C to T substitution at −1562 bp position (MMP9-1562C/T). For MMP13, a functional single-nucleotide polymorphism (SNP) has been detected -77A/G (rs2252070) in the MMP 13 promoter region, affecting its transcriptional activity [12,13]. A growing amount of evidence has reported that in different populations worldwide MMP9 -1562C/T or -77A/G polymorphisms may have a relationship with numerous cancers [14]. However, there was no definite conclusion on a relationship between these gene polymorphisms and the risk of lung cancer. Some studies have found that the C allele of genetic polymorphism in MMP-9 gene rs3918242 was significantly associated with an increased risk for the lung cancer patients, but the details on how genetic variant impacts MMP13 expression is still largely unknown. In recent years, there are contradictory results between the -77A/G polymorphisms in the MMP-13 and various types of cancer risk [15,16]. To investigate the role of the functional gene polymorphisms MMP9 -1562C/T (rs3918242) and MMP13 -77A/G (rs2252070) in the pathogenesis of certain diseases, we aim to evaluate the serum levels and polymorphisms of MMP-9 and MMP-13 in non-small cell lung cancer patients compared to normal subjects and their correlation to NCLC histopathology findings in Southern Chinese people.

## 2. Materials and Methods

### 2.1. Subjects

This study included 245 patients and 258 healthy individuals. The patients and controls were recruited in the Zhuzhou Central Hospital (Zhuzhou, China) and Hunan Cancer Hospital (Changsha, China) from January 2014 to March 2018.All of these individuals were unrelated ethnic Han Chinese.

The 245 patients with non-small cell lung cancer were diagnosed by cytology and histopathology were not receiving radiotherapy or chemotherapy, and unclear pathological diagnosis were eliminated; the histological classification and staging of all patients were performed by pathological evaluation and the clinical or pathological staged of lung cancer. The 258 controls was randomly recruited from healthy individuals who underwent routine physical examination in the same regions during the same period when the case patients were selected, which is healthy subjects with no history or prior diagnosis of any malignancy or any other serious disease around whom lungs was collected and used as control for this study. The control group individuals were age- and sex-matched with the case group individuals to the maximum extent possible. Each participant was interviewed to collect information on demographic characteristics. They completed detailed questionnaires including diet, smoking status, lifestyle, and medical history. The research protocol was approved by the Ethics Committee of Hunan Cancer Hospital on 27 April 2014,which was conducted according to the CFDA/GCP guidelines, regulatory requirements, and the provisions of the Helsinki Declaration, and all patients or their legal guardians signed informed consent statements.

### 2.2. Genomic DNA Extraction and Genotyping

#### 2.2.1. DNA Extraction

The venous blood samples and DNA samples were drawn in EDTA tube and stored at −70 °C. DNA was extracted from peripheral blood samples of patients and control subjects using the Ezup Column Animal Genomic DNA Purification Kit (Sangon Biotech Co., Ltd., Shanghai, China), following the manufacturer’s recommendations.

#### 2.2.2. MMP9 -1562C/T Polymorphism

**Figure 1:**
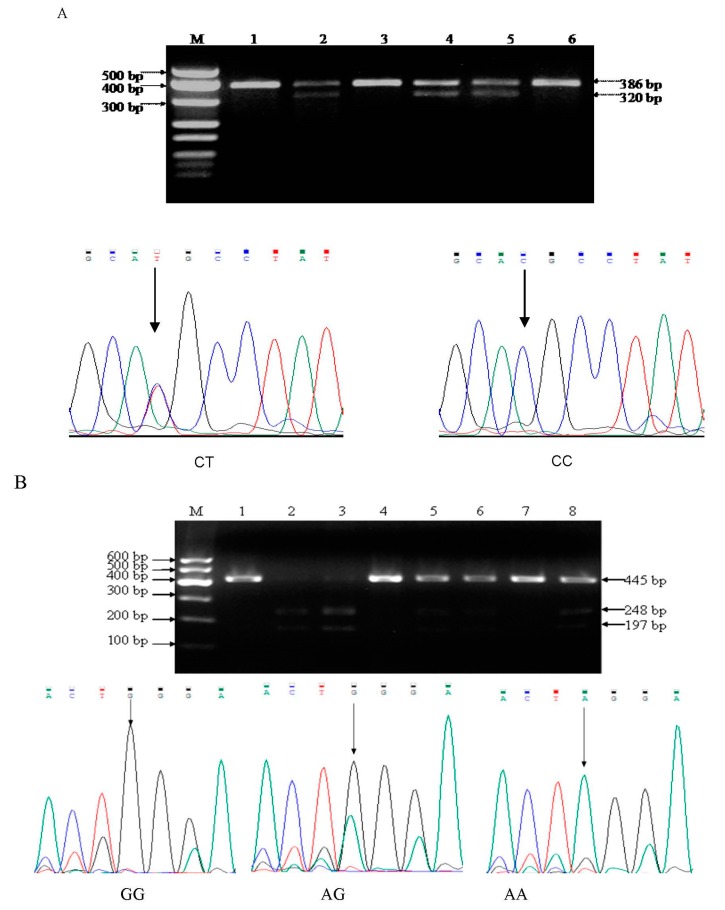
The polymerase chain reaction-restriction fragment length polymorphism (PCR-RFLP) analyses and direct-sequence result of MMP9 and MMP13 genes polymorphism. (**A**) MMP9 polymorphisms. Lanes 1, 3, and 6 CC homozygous genotype (386 bp) and lanes 2, 4, and 5, CT heterozygous genotypes (386, 320, and 66 bp). (**B**) MMP13 polymorphisms. Lanes 2 and 3, AA homozygous genotypes (248 and 197 bp); lanes 1, 4, and 7, GG homozygous genotypes (445 bp); and lanes 5, 6, and 8, AG heterozygous genotypes (445, 248, and 197 bp).

The polymorphisms MMP9-1562C/T were genotyped by polymerase chain reaction-restriction fragment length polymorphism (PCR-RFLP) using the primer pairs of forward: 5′-TGGTCAACGTAGTGAAACCCCATCT-3′; reverse: 5′-TCCAGCCCCAATTATCACACTTAT-3′ [17]. The PCR conditions used were 1 cycle at 95 °C for 2 min, followed by 40-step cycles of 94 °C for 30 s, 67 °C for 30 s, and 72 °C for 30 s, and a final extension step of 72 °C for 10 min [18]. Twenty units of PCR products were digested with SphI restriction enzyme (New England Biolabs, Beijing, LTD, China) for 1 h at 37 °C. The C allele was not cleaved by SphI, having a single 386 bp band, but the T allele was cut into two small fragments (320 and 66 bp); heterozygotes had a combination of both alleles (386 bp, 320 bp, and 66 bp bands). The genotyping by PCR-RFLP (Figure 1A) was confirmed by DNA sequencing; the results of PCR-RFLP genotyping and sequencing analysis were completely consistent.

#### 2.2.3. MMP13 -77A/G Polymorphism

The polymorphism in MMP13 was determined by PCR-RFLP, the oligonucleotide primers used for amplification of the specific promoter region containing the MMP13 -77A/G site were 5′-GATACGTTCTTACAGAAGGC-3′ (Forward) and 5′-GACAAATCATCTTCATCACC-3′ (Reverse) [19]. An initial denaturation step at 95 °C for 2 min was followed by 40 cycles of 94 °C for 30 s, 64 °C for 30 s, 72 °C for 30 s, and a final extension step at 72 °C for 10 min. Six units of Bsr I restriction enzyme (New England Biolabs, Inc.) was used to digest 10 μL PCR product for 2 h at 65 °C according to the instructions of the manufacturer. The G allele yields two bands of 248 bp and 197 bp, AG heterozygosity yields three bands—445, 248, and 197 bp—and the A allele yields a single 445 bp band. The genotyping by PCR-RFLP (Figure 1B) was confirmed by DNA sequencing; the results of PCR-RFLP genotyping and sequencing analysis were completely consistent.

### 2.3. Measurement of MMP-9 and MMP-13 ELISA

A total of 3 mL of blood was drawn and the serum separated and stored at −80 °C.

Serum levels of MMP-9 and MMP-13 levels were measured by using a specific ELISA, Human Matrix Metalloproteinase Enzyme Immunoassay Kits (Shanghai Enzyme Biotechnology Co., Ltd., Shanghai, China). Methods were as described in the manufacturer’s protocol. This 96-well sandwich immunoassay quantitatively determines the pro and active forms of MMP-9 and MMP-13 using a mouse anti-human MMP-9 and MMP-13 monoclonal antibody, biotinylated goat anti-human MMP-9, and an MMP-13 antibody, leading to yellow color development measured at 450 nm. All assays were performed in duplicate.

### 2.4. Statistical Analysis

Statistical analysis was carried out using the SPSS 23.0 software package (SPSS Company, Chicago, IL, USA). The categorical parameters (sex, age, smoking status, histological type, clinical stage, and lymph node metastasis) were compared by χ^2^-test and the continuous variables were compared by independent samples *t*-test or one-way ANOVA test. *p*-values less than 0.05 were considered statistically significant and all statistical tests were two-sided. The risk estimates were expressed as odds ratios (ORs) and 95% confidence intervals (95% CIs) and adjusted by age and gender accordingly.

## 3. Results

### 3.1. Characteristics of Study Subjects

The demographic and selected characteristics information of all the subjects is demonstrated in Table 1. The age and gender did not differ significantly between 245 lung cancer patients and 258 controls (*p* = 0.32 for age and *p* = 0.44 for sex). However, compared with the control subjects, the cases were more likely to be tobacco smokers (*p* < 0.05), which indicated that tobacco smoking was a high-risk factor for NSCLC in the present study population.

### 3.2. Distribution of MMP9 and MMP13 Polymorphisms

An unconditional logistic regression model was used to estimate the association between genotypes and the risk of developing NSCLC, and the genotype and allele frequencies of MMP9 and MMP13 polymorphisms are indicated in Table 2, Table 3 and Table 4 and Figure 1. The frequencies of CC, CT, and TT genotypes of the MMP9 were 82.04%, 17.96%, 0 and 67.05% in non-small cell lung cancer cases, and 67.05%, 32.95%, 0 in the control group, and the frequencies of GG, AG, and AA genotypes of the MMP13 were17.55%, 38.37%, 44.08% and 12.02% in patients, and 12.02%, and 33.72% and 54.26% in controls.

The multilayer relationship between MMP9 gene polymorphism distribution and clinic pathological parameters in non-small cell lung cancer and control group is shown in Table 2 and Table 3. The CC genotype of MMP9 -1562C/T SNPs showed no difference between cases and controls compared with TT+CT genotype in female (*p* > 0.05), and other groups were significantly different between cases and controls (*p* < 0.05). Moreover, there were no correlation between cases and controls with genotype of MMP-13-77A/G SNPs (*p* > 0.05).

The frequencies of the T allele of MMP9 -1562C/T SNPs and G allele of MMP13 -77A/G were significantly different between cases and controls (*p* < 0.05). By contrast, there was no difference in the frequency of the AG/GG allele of MMP-13-77A/G (*p* > 0.05).The results demonstrated a significant association between CT genotype and the T allele and a decreased risk of lung cancer in MMP9-1562C/T (*p* = 0.001, OR = 0.45, 95% CI = 0.29–68) compared with the CT+TT genotype; the C allele was associated with increased risk of developing lung cancer (*p* = 0.00, OR = 2.0, 95% CI =1.36–2.95) compared with that of the T allele in case–control research. Similarly, the MMP13 AA genotype might be a marker of decreased genotype susceptibility to lung cancer compared with the GG genotype (*p* = 0.03, OR = 0.56, 95% CI = 0.33–0.94). In additional, there was a significant association between the A allele and a decreased risk of lung cancer, compared with the G allele (*p* = 0.00, OR = 0.70, 95% CI = 0.54–0.91). The result showed that significantly different frequencies among subjects carrying both the MMP9 CC and MMP13 GG genotypes in cases and controls. Moreover, MMP9 gene was deviated from Hardy–Weinberg equilibrium in controls (*p* < 0.01), other groups were in-line with Hardy–Weinberg equilibrium in the patient and control groups (*p* > 0.05), and the results were shown in Table 5.

### 3.3. The Association between MMP Polymorphisms and NSCLC Risk

An unconditional logistic regression model was used to estimate the association between genotypes of MMP9 and MMP13 and the risk of lung cancer in Table 6 and Table 7. The results demonstrated no difference was founded between MMP9 polymorphisms and NSCLC with histological type, clinical stage, and lymph node metastasis parameters (*p* > 0.05), and there was no difference in clinical stage with MMP13 polymorphism (*p* > 0.05), but a significant association with histological type, clinical stage, and lymph node metastasis (*p* < 0.05).

These subjects were also analyzed whether there was a statistical joint effect between the MMP9 and MMP13 polymorphisms in Table 8. The result showed that a significantly different frequencies among subjects carrying both MMP9 CC and MMP13 GG genotypes in cases and controls (14.29% vs. 3.67%). Moreover, the object of carrying MMP9 CC and MMP13 GG genotypes along showed a significantly increased risk of lung cancer (*p* = 0.00, OR = 5.34, 95% CI = 2.46–11.60) compared with other genotypes.

### 3.4. Distribution of with Serum Levels MMP and Polymorphisms

In this study, 67 patients with NSCLC and 21 health subjects were included. The NSCLC and control groups were randomly extracted according to the ratio of Table 1. The NSCLC group had a significantly higher serum level of MMP-9 (194.05 ± 17.56 vs. 45.21 ± 14.52 ng/mL, *p* = 0.00) and MMP-13 (288.16 ± 8.97 vs. 57.56 ± 7.95 ng/mL; *p* < 0.001) compared to healthy subjects.

The genotype and allele of MMP9 -1562C/T SNPs and MMP13-77A/G were not significantly different between cases and controls (*p* > 0.05), and the results demonstrated an association between the C allele and an increased serum level of NSCLC in MMP9-1562C/T (OR = 1.19, 95% CI = 0.75–1.89) compared with the A allele. Similarly, the AA genotype of MMP13 might be a marker of decreased serum level of NSCLC (OR = 0.76, 95% CI = 0.51–1.14), and the results were shown in Table 9.

## 4. Discussion

Several polymorphisms in the MMP genes have been reported for their possible relation to varieties malignant tumors [15,18,20]. The present study is a case–control study aimed to demonstrate the effects of the association between the MMP9 -1562C/T (rs3918242) and MMP13 -77A/G (rs2252070) gene polymorphisms, alone or under interaction, and the risk of lung cancer in our Chinese Population. Our observations were supported the hypothesis that genetic polymorphisms of MMP13 and MMP9 might function as key oncogenes in lung carcinogenesis.

MMP9 might be the most important in tumor invasion and metastasis, especially in prostate cancer [21]. An overview of MMP9 polymorphism and gastric cancer risk by Verma et al. identified that MMP9 -1562 C/T could be an important SNP for increased expression of MMP9 in a particular locality [22]. A research by Avcı et al. found that there were no significant differences between subjects with the CC and CT+TT genotypes in the pathogenesis and clinical course of GC in Turkish subjects [23]. Similarly, Alicia et al. indicated that common genetic variation in MMP9 was not significantly associated with altered breast cancer susceptibility among participants of the shanghai breast cancer genetics study [24]. Matsumura et al. demonstrated that the T allele in the MMP9 promoter was associated with the invasive phenotype of gastric cancer [25]. Contrary to these findings, a recent case–control study and a minireview by Banday et al. found that the CT heterozygous genotype of MMP9-1562C/T SNP showed a significant association with increased risk for the development of colorectal cancer in Kashmiri population, compared with T allele [5]. Differently, our previous studies found that compared with the CT genotype, the MMP9-1562CC genotype might be a marker of increased genotype susceptibility to NSCLC among the South-Central Chinese population [19]. Although the genotypes were evenly distributed between NSCLC patients and healthy individuals in current research, no difference were founded between MMP9 polymorphisms and NSCLC with histological type, clinical stage, lymph node metastasis parameters (*p* > 0.05). Some promising results were obtained, which showed that subjects carrying the T allele were at a decreased risk of lung cancer in MMP9-1562C/T, and the C allele was associated with increased risk of developing lung cancer. Moreover, CC genotypes were found at much higher frequency than TT genotypes between NSCLC patients and controls. We also found that the serum levels of MMP9 were detected in NSCLC and controls. The C allele of MMP9 showed a higher expression than the T allele. Our dataset further proved that the serum levels of MMP-9 with NSCLC were compared with the obvious increase in the control group (*p* < 0.05), the C allele of MMP-9 showed a higher expression s than in T allele, the serum levels of MMP-9 in peripheral blood can reflect the load state of the body, and a large number of scientific studies have shown that high expression of MMP-9 is closely related to tumorigenesis, invasion and metastasis, and patient prognosis [12,17,19,26], and there is a correlation between genetic findings and enzymatic activity, the results demonstrated that C allele of MMP9-1562C/T could increase serum level of NSCLC compared with the A allele (OR = 1.19, 95% CI = 0.75–1.89) and CC genotype, and the C allele could increase the risk of lung cancer, which was consistent with the results of the SNP analysis.

The CC genotypes of MMP9-1562C/T should be associated with the increased risk of developing NSCLC with Southern Chinese people.

In the MMP13 promoter, Yoon et al. found a polymorphic variant as an A to G transition at position -77 (MMP13 -77A/G). The excessive expression of MMP13 has been demonstrated to associate with poor survival in various malignant tumors [12,26,27,28,29]. Elevated MMP13 expression was not only observed in cancer tissues but also associated with tumor invasion, vascular permeation, and lymph node metastasis [28]. MMP-13 is shown to be overexpressed in varieties of tumors, which implies that a high level of MMP-13 may be closely related to tumor invasion, metastasis, and poor prognosis in several forms of cancer [30,31]. The research by Li et al. not only confirmed the association between MMP13 polymorphisms and lung cancer risk in a recessive model, but also with a model-free approach [3]. Similarly, studies performed by Vairaktaris et al. analyzing association of MMP-13 with oral cancer indicated that MMP-13 is not a major contributing factor for the initiation of oral cancer [31]. Contrary to these findings, Martin et al. found that the AA genotype of the MMP13 (-77 A/G) SNP was significantly more frequent in sepsis development and outcome in ICU patients [32]. Moreno et al. demonstrated that the MMP13 A allele was found to be approximately two times higher than the G allele in the same position [10].

In present, no difference were founded between MMP13 polymorphisms between NSCLC and controls (*p* > 0.05), The frequencies of the G allele of MMP13 -77A/G were significantly different between cases and controls (*p* < 0.05), and the MMP13 AA genotype might be a marker of decreased genotype susceptibility to NSCLC compared with the GG genotype (*p* < 0.05). In additional, the A allele was found to be higher than the G allele. Moreover, the serum levels of MMP13 were detected between NSCLC and controls. Some scientific researchers reported that high expression of MMP13 was associated with NSCLC infiltration, metastasis, and recurrence [12,17,19,26,31]. The G allele of MMP13 showed a higher expression than the A allele in our experiment. Similarly, the AA genotype of MMP13 might be a marker of decreased serum level of lung cancer, which could decrease the risk of lung cancer and be consistent with the results of the SNP analysis. The A allele and AA genotype of MMP13-77 may protect against lung cancer, and should have an associated with a decreased risk of NSCLC with Southern Chinese people. Moreover, there was a statistical joint effect between the MMP9 and MMP13 polymorphisms in NSCLC patients and controls; significantly different frequencies were founded among subjects carrying both the MMP9 CC and MMP13 GG genotypes in cases and controls (14.29% vs. 3.67%), the subject of carrying MMP9 CC and MMP13 GG genotypes showed a significantly increased risk of NSCLC (*p* < 0.01, OR = 5.34) compared with other genotypes. and there may be gene–gene interactions.

Our data demonstrated evident compliance between genetic findings and enzymatic activity; however this correlation is still only a trend. Because the occurrence and development of tumors are affected by many factors [12,17,19,31], and the single-nucleotide polymorphism (SNP) depends on the genetic background of the individual as well as environmental factors, a limitation of the present study was the small number of patients with each category of gene type, which may affect the estimates of results for genotype, and some further scientific researchers should be needed in future.

## 5. Conclusions

According to our findings, the subject of carrying MMP9 CC and MMP13 GG genotypes showed a significantly increased risk of NSCLC, and the serum levels of MMP9 and MMP13 constitute statistical evidence in support of the notion that MMP13 and MMP9 might function as a key oncogene in NSCLC with Southern Chinese population. Combined detection of SNP and enzyme activity between MMP9 and MMP13 is expected to be a potential diagnostic method for non-small cell lung cancer.

## Figures and Tables

**Table 1:** Demographic characteristic of lung cancer cases and controls.

Variable	Cases (*n* = 245) Number (%)	Controls (*n* = 258) Number (%)	*p*-Value	χ^2^
Age			0.32	0.97
<55	100 (40.82)	118 (45.74)		
≥55	145 (59.18)	140(54.36)		
Sex			0.44	0.61
Male	180 (73.47)	165 (63.95)		
Female	65(27.53)	93 (36.05)		
Smoking status			<0.001	38.61
No	85 (34.69)	161 (62.40)		
Yes	160 (65.31)	97 (37.60)		
Histological type				
Squamous carcinomas	135 (55.11)			
Adenocarcinomas	89(36.32)			
Other carcinomas ^a^	21 (8.57)			
Clinical stage I + II	80			
III + IV	165			
Lymph node metastasis				
Yes	182			
No	63			

^a^ including the small cell, large cell, and mixed cell carcinomas or undifferentiated carcinomas.

**Table 2:** Associations with MMP9 polymorphisms in non-small cell lung cancer (NSCLC) and control groups.

Variable	Genotype	Cases (*n* = 245) Number (%)	Controls (*n* = 258) Number (%)	*p*	χ^2^	OR (95% CI) ^b^
Age						
<55	CC	81 (81.00)	80 (67.80)	0.03	4.89	2.03 (1.08–3.81) ^c^
	TT+CT	19 (19.00)	38 (32.20)			1 (reference)
≥55	CC	120 (82.75)	93 (66.43)	0.002	10.06	2.43 (1.39–4.23) ^c^
	TT+CT	25 (17.25)	47 (33.57)			1 (reference)
Sex						
Male	CC	148 (82.22)	104 (63.03)	0.00	16.10	2.71 (1.65–4.45) ^c^
	TT+CT	32 (17.78)	61 (36.97)			1 (reference)
Female	CC	53 (81.54)	69 (74.19)	0.28	1.17	1.54 (0.70–3.35)
	TT+CT	12 (18.56)	24 (26.81)			1 (reference)
Smoking status						
Yes	CC	132 (82.50)	66 (68.04)	0.01	7.14	2.21 (1.23–4.00) ^c^
	TT+CT	28 (17.50)	31 (31.96)			1 (reference)
No	CC	69 (81.18)	107 (66.46)	0.02	5.92	2.18 (1.15–4.11) ^c^
	TT+CT	16 (18.82)	54 (33.54)			1 (reference)

^b^ ORs was adjusted for age, sex, cigarette smoking, and alcohol consumption. *p*-value: Pearson χ^2^ or Fisher exact test two-sided value, ^c^ *p* < 0.05. OR, odds ratio; CI, confidence interval.

**Table 3:** Associations with MMP13 polymorphisms in NSCLC and control groups.

Variable	Genotype	Cases (*n* = 245) Number (%)	Controls (*n* = 258) Number (%)	*p*	χ^2^	OR (95% CI) ^b^
Age						
<55	GG	25 (17.24)	17 (12.14)			1 (reference)
	AA + AG	120 (82.76)	123 (87.86)	0.23	1.47	1.51 (0.78-2.93)
≥55	GG	18 (18.00)	14 (11.86)			1 (reference)
	AA + AG	82 (82.00)	104 (88.14)	0.20	1.63	1.63 (0.77–3.47)
Sex						
Male	GG	31 (17.22)	20 (12.12)			1 (reference)
	AA + AG	149 (82.78)	145 (87.82)	0.18	1.78	1.51 (0.82–2.77)
Female	GG	12 (18.46)	11 (11.83)			1 (reference)
	AA + AG	53 (81.54)	82 (88.17)	0.25	1.35	1.69 (0.69–4.10)
Smoking status						
Yes	GG	30 (18.75)	13 (13.40)			1 (reference)
	AA + AG	130 (81.25)	84 (86.60)	0.26	1.24	1.49 (0.74–3.02)
No	GG	13 (15.29)	18 (11.18)			1 (reference)
	AA + AG	72 (84.71)	143 (88.82)	0.36	0.86	1.43 (0.67–3.09)

^b^ ORs was adjusted for age, sex, cigarette smoking, and alcohol consumption. *p*-value: Pearson χ^2^ or Fisher exact test two-sided value. OR, odds ratio; CI, confidence interval.

**Table 4:** Allele frequencies of MMP-9 and MMP-13 among cases and controls.

Genotype	Cases (*n* = 245)	Controls (*n* = 258)	*p*-Value	χ^2^	OR (95% CI) ^b^
MMP-9					
CC/CC	201 (82.04)	173 (67.05)	-	-	1 (reference)
CT/CT	44 (17.96)	85 (32.95)	0.00	14.80	0.45 (0.29–0.68) ^c^
TT/TT	0	0			
T allele frequency	44 (9.98)	85 (16.47)	-	-	1 (reference)
C allele frequency	446 (91.02)	431 (83.53)	0.00	12.62	2.0 (1.36–2.95) ^c^
MMP-13					
GG/ GG	43 (17.55)	31 (12.02)	-	-	1 (reference)
AG/ GG	94 (38.37)	87 (33.72)	0.37	0.81	0.78 (0.45–1.35)
AA/ AA	108 (44.08)	140 (54.26)	0.03	4.85	0.56 (0.33–0.94) ^c^
G allele frequency	180 (36.73)	149 (28.88)	-	-	1 (reference)
A allele frequency	310 (63.26)	367 (71.12)	0.00	7.05	0.70 (0.54–0.91) ^c^

^b^ ORs was adjusted for age, sex, cigarette smoking, and alcohol consumption. *p*-value: Pearson χ^2^ or Fisher exact test two-sided value, ^c^ *p* < 0.05. OR, odds ratio; CI, confidence interval.

**Table 5:** Allelic frequency and logistic regression analysis data of MMP-9 and MMP-13.

Gene	Group	Alleles		*p*-Value (HWE Test)	χ^2^
		T	C		
MMP9	Case	0.09	0.91	0.33	2.2
	control	0.18	0.82	0.01	8.50
MMP13		G	A		
	Case	0.42	0.66	0.12	4.18
	control	0.35	0.74	0.06	5.81

*p*-Value: Pearson χ^2^ or Fisher exact test two-sided value.

**Table 6:** Correlation between MMP9 and clinical pathology parameters in NSCLC.

Variable	N	Genotype			*p*	χ^2^
		CC	CT	TT		
Histological type					0.82	0.66
Squamous carcinomas	135	113 (82.22)	22 (17.78)	0		
Adenocarcinomas	89	72 (80.90)	17 (19.10)	0		
Other carcinomas ^a^	21	16 (76.19)	5 (23.81)	0		
Clinical stage						
I + II	80	70 (87.50)	10 (12.50)	0	0.12	2.40
III + IV	165	131 (79.39)	34 (20.61)	0		
Lymph node metastasis						
Yes	182	150 (82.42)	32 (17.58)	0	0.79	0.068
No	63	51 (80.95)	12 (19.05)	0		

^a^ including the small cell, large cell, and mixed cell carcinomas or undifferentiated carcinomas. Value: Pearson χ^2^ or Fisher exact test two-sided value.

**Table 7:** Correlation between MMP-13 and clinical pathology parameters in NSCLC.

Variable	N	Genotype			*p*	χ^2^
		GG	AG	AA		
Histological type					0.00	22.70
Squamous carcinomas	135	11 (8.15)	59 (43.70)	65 (48.15)		
Adenocarcinomas	89	23 (25.84)	28 (31.46)	38 (42.70)		
Other carcinomas ^a^	21	9 (42.86)	7 (33.33)	5 (23.81)		
Clinical stage					0.126	4.15
I + II	80	17 (21.25)	31 (38.75)	32 (40.00)		
III + IV	165	26 (15.76)	63 (38.18)	76 (46.06)		
Lymph node metastasis					0.00	64.8
Yes	182	28 (15.38)	72 (39.56)	82 (45.06)		
No	63	15 (23.81)	22 (34.92)	26 (41.27)		

^a^ including the small cell, large cell, and mixed cell carcinomas or undifferentiated carcinomas. Value: Pearson χ^2^ or Fisher exact test two-sided value.

**Table 8:** Risk of lung cancer in association with MMP9 and MMP13 polymorphism.

Genotype		Cases n (%) *n* = 245	Controls n (%) *n* = 258	*p*	χ^2^	OR (95% CI)
MMP9	MMP13					
TT+CT	AA+AG	35 (14.29)	66 (25.58)	-		1 (reference)
TT+CT	GG	9 (3.67)	19 (7.36)	0.80	0.06	0.89 (0.37–2.18)
CC	AA+AG	167 (68.16)	161 (62.41)	0.00	8.20	1.96 (1.23–3.11)
CC	GG	34 (13.88)	12 (4.65)	0.00	19.56	5.34 (2.46–11.60)

**Table 9:** The association with serum levels and matrix metalloproteinase (MMP) genotype in NSCLC and control.

Genotype	Cases (*n* = 67)	Controls (*n* = 21)	*p*-Value	χ^2^	OR (95% CI) ^b^
MMP-9					
CC/CC	61 (198.36 ± 25.21)	14 (46.55 ± 15.78)	-	-	1 (reference)
CT/CT	6 (154.74 ± 22.35)	7 (42.53 ± 14.26)	0.51	0.43	0.86 (0.54–1.36)
TT/TT	0	0			
T allele	6 (154.74 ± 21.71)	7 (42.53 ± 14.79)	-	-	1 (reference)
C allele	128 (196.58 ± 25.71)	35 (45.75 ± 15.36)	0.468	0.526	1.19 (0.75–1.89)
MMP-13					
GG/ GG	31 (343.41 ± 37.28)	10 (56.68 ± 18.17)	-	-	1 (reference)
AG/ GG	29 (227.29 ± 35.27)	7 (55.96 ± 17.85)	0.06	3.68	0.67 (0.45–1.01)
AA/ AA	7 (295.64 ± 41.63)	4 (62.61 ± 19.32)	0.25	1.34	0.79 (0.53–1.17)
G allele	91 (306.41 ± 45.39)	27 (56.49 ± 18.29)	-	-	1 (reference)
A allele	43 (249.54 ± 19.57)	15 (59.50 ± 18.55)	0.18	1.77	0.76 (0.51–1.14)

Value: Pearson χ^2^ or Fisher exact test two-sided value, ^b^ *p* < 0.05.
